# Uptake and determinants of Hepatitis B vaccination among health laboratory practitioners in tertiary hospitals in Dar es Salaam, Tanzania: A cross-sectional study

**DOI:** 10.1371/journal.pgph.0005662

**Published:** 2026-04-27

**Authors:** Seleman Said, Mary Migiro, Dominic Renatus, Raidah R. Gangji, Lilian Nkinda, Upendo Kibwana, Frank Msafiri, Doreen Kamori, Agricola Joachim, Joel Manyahi, Mtebe V. Majigo, Salim S. Masoud

**Affiliations:** 1 Muhimbili University of Health and Allied Sciences, Department of Microbiology and Immunology, Dar es Salaam, Tanzania; 2 Central Pathology Laboratory, Muhimbili National Hospital, Dar es Salaam, Tanzania; PLOS: Public Library of Science, UNITED STATES OF AMERICA

## Abstract

Hepatitis B virus (HBV) infection is a significant occupational risk for laboratory practitioners. Despite proven vaccine effectiveness and global recommendations, HBV vaccine uptake among healthcare workers in Tanzania remains low. This study assessed HBV vaccination uptake and its determinants among laboratory practitioners in tertiary hospitals in Dar es Salaam. An analytical cross-sectional study was conducted from March to June 2025 across four tertiary hospitals, enrolling 130 participants using the Kish-Leslie formula. Data were collected using a structured, pre-tested, interviewer-administered questionnaire and verified vaccination records, and analyzed with IBM SPSS v27. Descriptive statistics summarized participant characteristics, and associations were assessed using chi-square, Fisher’s exact tests, and log-binomial regression with robust variance. Of 130 participants (median age 30 years, IQR: 25–36), 54.6% (71/130) completed the three-dose hepatitis B vaccination schedule, 23.8% (31/130) received partial doses, and 21.5% (28/130) were unvaccinated. Among the partially vaccinated, missed appointments (48.4%, 15/31) and lack of vaccine availability (41.9%, 13/31) were the main reasons. Half of the unvaccinated (50.0%, 14/28) cited lack of opportunity. Most participants knew the vaccine is essential (99.2%, 129/130), and 94.1% (96/102) acknowledged three doses are required for full protection. Although females, those with longer work experience, and those perceiving high exposure risk had higher vaccination prevalence, none of these associations reached statistical significance in univariate or multivariable analyses. HBV vaccine uptake among laboratory practitioners in Dar es Salaam is suboptimal, mainly due to structural barriers. Strengthening workplace vaccination programs, ensuring consistent vaccine supply, and implementing reminder systems could improve healthcare worker protection.

## Introduction

Hepatitis B virus (HBV) infection continues to be a significant concern for public health worldwide and a leading contributor to illness and death, especially in resource-limited countries [1,2]. The World Health Organization (WHO) estimated 257 million people were living with chronic HBV infection in 2022, which resulted in 1.1 million deaths worldwide. HBV infection can lead to severe liver disorders such as liver cirrhosis and hepatocellular carcinoma [3]. Due to the increased rate of HBV infection and the worry for the world’s public health, the WHO developed a global viral hepatitis strategy in 2016 for the elimination of HBV infection by the year 2030. The strategy aimed at reducing new infections by 90% and the death rate by 65% [4,5]. In addressing viral hepatitis as part of the global health agenda under the 2030 Sustainable Development Goals (particularly SDG 3.3), the World Health Organization developed a Global Health Sector Strategy on Viral Hepatitis that outlines elimination targets and priorities aligned with the SDGs (WHO, 2016) [6]. As part of this global framework, the United Republic of Tanzania adopted a National Strategic Plan for the Prevention, Control, and Elimination of Viral Hepatitis 2018/19–2022/23, which prioritizes prevention, surveillance, and control of viral hepatitis. This plan specifically recommends hepatitis B vaccination for all healthcare workers as a key strategy to reduce occupational transmission and strengthen health systems [7].

According to WHO estimates, vaccination coverage among HCWs in countries with low- and moderate-income levels ranges from 18% to 39%, compared to 67% to 79% in high-income countries [8,9]. In Tanzania, the proportion of Hepatitis B vaccination coverage among HCWs ranges from 18.9 to 70.5% [9]. Among HCWs, laboratory practitioners represent a subgroup at especially high risk for occupational exposure to bloodborne pathogens, including HBV, due to frequent handling of blood specimens, sharps, and contaminated materials [10–12]. Many studies on hepatitis B vaccination among healthcare workers report aggregated data, with limited breakdown by professional role. Where disaggregated data exist, non-physician cadres, including laboratory staff, have been shown to have significantly lower likelihood of vaccination. For example, laboratory personnel in Ethiopia and other African settings had adjusted odds ratios ranging from 0.19–0.47 compared with physicians, highlighting a substantial disparity in uptake across professional categories [13,14]. In Nigeria, hepatitis B vaccination uptake among healthcare workers has been consistently low despite high awareness of occupational risk. In a cross-sectional study of HCWs in two teaching hospitals, only 36.2% of participants were fully vaccinated, and professional category (including laboratory scientists) was significantly associated with vaccination status, with laboratory staff more likely to be unvaccinated [15].

Despite the introduction of the hepatitis B vaccination in Tanzania in 2002, vaccination coverage among HCWs remains suboptimal, with significant regional disparities and inadequate understanding of the barriers to vaccination. For example, studies have reported full vaccination rates ranging from 18.9% in primary facilities to 33.6% in tertiary hospitals and 67.4% of HCWs having received at least one dose in northern Tanzania [9,16,17]. This raises concerns about the potential risks of HBV transmission in healthcare settings and highlights the need for targeted interventions to improve vaccination rates. Therefore, in view of that, this study was designed to assess hepatitis B vaccination uptake and its predicting factors among healthcare laboratory practitioners working in the tertiary hospitals in Dar es Salaam.

## Materials and methods

### Ethics statement

This study was approved by the Institutional Review Board of Muhimbili University of Health and Allied Sciences (Ref.No.DA.282/298/01.C/2568). Permission to conduct the study was obtained from the respective hospital administrations. Before registration in the study, informed consent was obtained from all participants.

### Study design and setting

An analytical cross-sectional study was conducted between March and June 2025 across four tertiary hospitals in Dar es Salaam, Tanzania: Amana Regional Referral Hospital, Mwananyamala Regional Referral Hospital, Temeke Regional Referral Hospital, and Muhimbili National Hospital. The study was reported in accordance with the Strengthening the Reporting of Observational Studies in Epidemiology (STROBE) guidelines for cross-sectional studies. Amana Regional Referral Hospital, serving the Ilala Municipality with approximately 1.2 million residents, operates with around 362 authorized beds and manages between 800 and 1,200 outpatient visits daily. Mwananyamala Regional Referral Hospital serves as the referral center for Kinondoni, Ubungo, and surrounding areas, covering a catchment population of over 2.2 million people. Temeke Regional Referral Hospital serves the Temeke District, which has a population of approximately 1.2 million and currently operates with 304 beds. Finally, Muhimbili National Hospital (MNH), the national referral, teaching, and research hospital, provides services to patients from all over Tanzania and neighboring countries, boasting a robust infrastructure with 1,500 beds. These hospitals were selected not only because they have a relatively higher number of laboratory workers compared to other facilities, but also because they provide a representative mix of regional referral and national hospital settings in Dar es Salaam.

### Study population

The study participants included laboratory practitioners working in clinical diagnostic laboratories at four selected hospitals. These practitioners consisted of laboratory technologists, scientists, and laboratory assistants who were actively involved in sample collection, processing, analysis, and reporting laboratory results. To qualify, participants had to provide written informed consent and verifiable hepatitis B vaccination records, such as a vaccination card. We excluded individuals who refused to participate or claimed to have received a hepatitis B vaccination without providing proof.

### Sample size and sampling technique

The minimum sample size was calculated using Kish-Leslie’s formula for a single population proportion:


$$n=\frac{Z^{\text{2}}p(\text{1}-p)}{d^{\text{2}}}$$

where Z=1.96  corresponding to a 95% confidence level, p=0.70  based on previous estimates of hepatitis B vaccination coverage among healthcare workers at Kilimanjaro Christian Medical Center in 2021 [17], and d=0.08  representing the margin of error.

Substituting these values:


$$n=\frac{\left(\text{1}.\text{9}\text{6}{)}^{\text{2}}\times \text{0}.\text{7}\text{0}\times (\text{1}-\text{0}.\text{7}\text{0}\right)}{(\text{0}.\text{0}\text{8}{)}^{\text{2}}\;}$$

$$n=\frac{\text{3}.\text{8}\text{4}\text{1}\text{6}\times \text{0}.\text{7}\text{0}\times \text{0}.\text{3}\text{0}}{\text{0}.\text{0}\text{0}\text{6}\text{4}}$$

$$n=\frac{\text{0}.\text{8}\text{0}\text{6}\text{7}}{\text{0}.\text{0}\text{0}\text{6}\text{4}}=\text{1}\text{2}\text{6}$$

**Fig 1:**
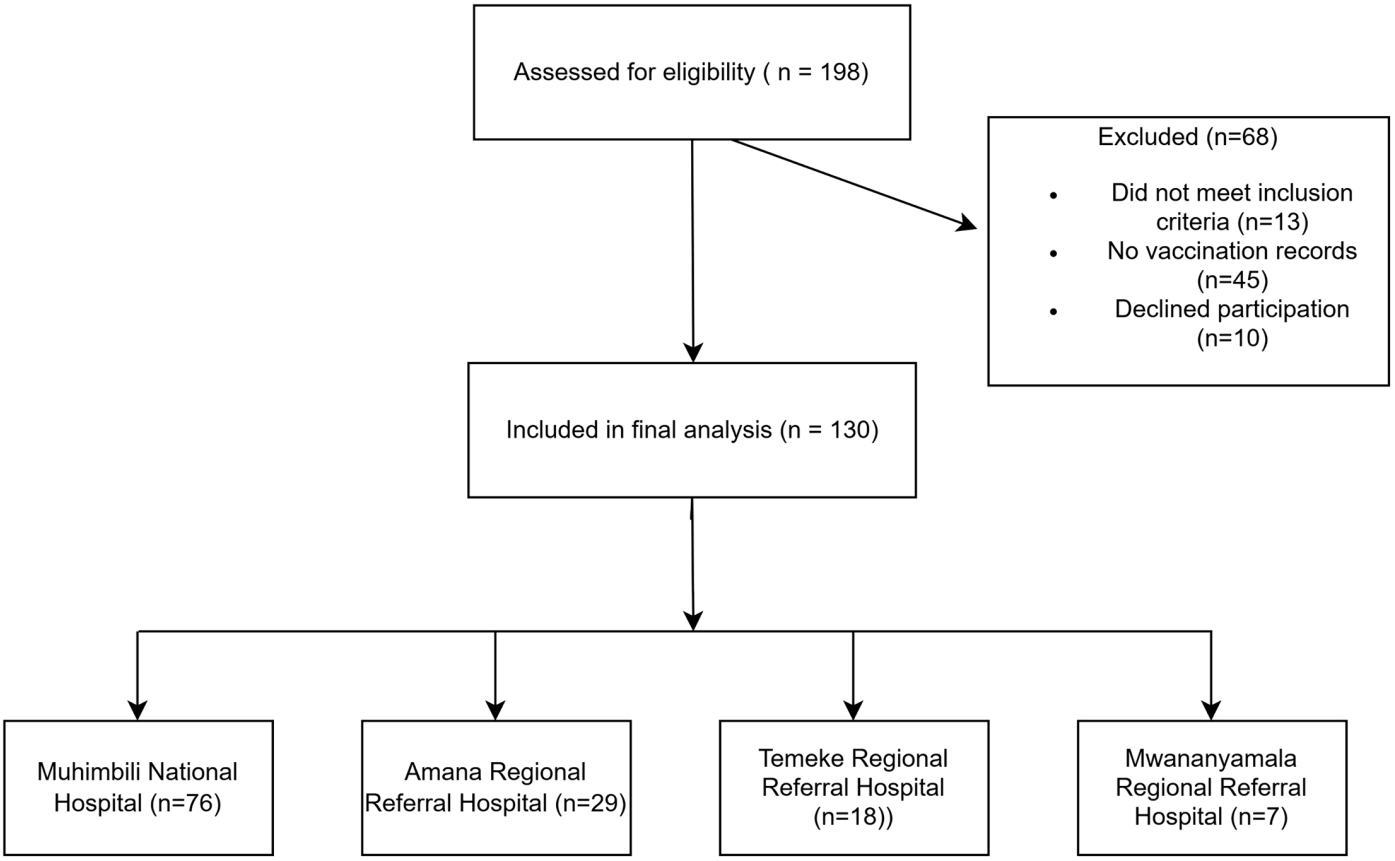
Participant recruitment flow diagram for health laboratory practitioners in four tertiary hospitals in Dar es Salaam, Tanzania.

The calculated minimum sample size was therefore 126 participants, after adjusting for potential non-response (3%), the final minimum required sample size was 130 participants. A 3% non-response rate was assumed based on expected high participation in a targeted, workplace-based survey of laboratory practitioners, direct distribution and follow-up. Probability proportional to size (PPS) sampling was then used to allocate the total sample (n = 130) across the four hospitals based on their estimated laboratory practitioner populations (Muhimbili: 177; Amana: 32; Temeke: 28; Mwananyamala: 30; total = 267). The target allocations were Muhimbili (86), Amana (15), Temeke (14), and Mwananyamala (15). Within each hospital, consecutive convenience sampling was applied. Due to variations in staff availability and response rates across sites, the final recruited numbers differed from the PPS targets (Muhimbili: 76, Amana: 29, Temeke: 18, Mwananyamala: 7). These deviations from the PPS targets reflect practical field realities and did not impact the overall sample size required for analysis. The participant recruitment process is summarized in Fig 1.

### Data collection

Data were collected using a validated structured questionnaire designed to capture relevant information from participants. The questionnaire was digitalized using Google Forms before the main data collection. The tool was pilot tested on 13 participants to assess clarity and functionality, and necessary adjustments were made to ensure data quality. Verbal consent was obtained before distributing the private Google Form link. The Google form link also contained a detailed written informed consent document before participants could participate in the study.

We also collected information on participants’ knowledge of hepatitis B vaccine effectiveness, number of recommended doses, safety, and occupational exposure risk, which was assessed using four separate questions in the structured questionnaire. For vaccine effectiveness, participants who responded, “very effective” were awarded 1 point, while responses of “less effective,” “slightly effective,” or “I don’t know” were scored 0. For vaccine safety, the correct response was “yes,” scored as 1 point, while “no” was scored 0. For occupational exposure risk, participants selecting “high risk” were awarded 1 point, while “low risk,” “moderate risk,” or “I don’t know” were scored 0. For knowledge on the number of recommended doses, the correct answer “three doses” awarded 1 point, while responses “two doses” or “one dose” were scored 0. For analytical purposes, perception-related variables were dichotomized. For vaccine effectiveness, responses were categorized into “very effective” and “not very effective,” which combined “slightly effective,” “less effective,” and “I don’t know”. For occupational exposure risk, “high risk” was categorized as correct recognition, while “moderate risk,” “low risk,” and “I don’t know” were grouped as lower perceived risk.

This categorization was applied to improve model stability, enhance interpretability of odds ratios, and avoid small cell counts that could compromise multivariable regression estimates. These binary scores contributed to the overall knowledge score used in the analysis.

### Statistics

Data were analyzed using IBM SPSS Statistics version 27.0. Categorical variables were summarized as frequencies and percentages, while continuous variables such as age were presented as medians with interquartile ranges (IQR). The relationship between independent variables and hepatitis B vaccination uptake was assessed using the Chi-square and Fisher’s exact tests. To further determine the strength and direction of associations while controlling for potential confounders, log-binomial regression with robust variance estimation was employed to calculate crude and adjusted prevalence ratios (cPR and aPR), along with corresponding 95% confidence intervals, in both univariate and multivariate analyses. A p-value of <0.05 was considered statistically significant.

Both statistical and epidemiological considerations guided the selection of variables for the multivariable analysis. Variables with a p-value less than 0.20 in univariate analysis were considered for inclusion, and those recognized in previous literature as potential confounders were also retained. In addition, age group and work experience were included a priori due to their theoretical relevance to both exposure and outcome, thereby ensuring adequate adjustment and reducing the risk of residual confounding. Model performance was assessed using the Omnibus Test of Model Coefficients and the Nagelkerke R² statistic.

## Results

### Demographic and professional characteristics

Of the 130 participants enrolled, 57.7% (75/130) were male, and the median age was 30 years, with an interquartile range (IQR) of 25–36 years. Most participants, 54.3% (25/46) were aged below 40 years. The majority were from MNH, comprising 58.5% of participants (76/130). The microbiology and phlebotomy sections were the most represented, each with 19.2% (25/130). Additionally, 81.5% (106/130) of participants had more than two years of work experience. Most respondents perceived themselves to be at high risk of HBV infection, 76.9% (100/130). Most participants believed the HBV vaccine to be very effective, 76.2% (99/130), and similarly, the vaccine was perceived as safe by 87.7% (114/130) of respondents. Nearly all participants, 99.2% (129/130), acknowledged the importance of the HBV vaccine for the healthcare workers “Table 1”.

**Table 1:** Proportion of hepatitis B vaccination uptake among health laboratory practitioners at referrals and tertiary hospitals in Dar es Salaam.

Variable	Total *n* (%) *	Vaccination uptake	P-value
		Yes, *n* (%)	
**Number of shots**			
Complete	71 (54.6)		
Incomplete	31 (23.8)		
Not received any shot	28 (21.5)		
**Sex**			0.077
Male	75 (57.7)	36 (48.0)	
Female	55 (42.3)	35 (63.6)	
**Median age (IQR)**	30 (25-36)	30 IQR (26–36)	0.099
**Age group (years)**			0.143
20-29	22 (16.9)	16 (72.7)	
30-39	46 (35.4)	25 (54.3)	
40+	62 (47.7)	30 (48.4)	
**Facility**			0.777
Muhimbili National Hospital	76 (58.5)	40 (52.6)	
Amana Regional Referral Hospital	29 (22.3)	17 (58.6)	
Temeke Regional Referral Hospital	18 (13.8)	9 (50.0)	
Mwananyamala Regional Referral Hospital	7 (5.4)	5 (71.4)	
**Laboratory section**			0.454
Phlebotomy	25 (19.2)	12 (48.0)	
Hematology	23 (17.7)	15 (65.2)	
Microbiology	25 (19.2)	16 (64.0)	
B/ transfusion	14 (10.8)	7 (50.0)	
Chemistry	12 (9.2)	5 (41.7)	
Serology	9 (6.9)	6 (66.7)	
Parasitology	12 (9.2)	6 (50.0)	
Histology	6 (4.6)	1 (16.7)	
Others	4 (3.1)	3 (75.0)	
**Work experience (years)**			0.120
Less than 2 years	24 (18.5)	7 (29.2)	
More than 2 years	106 (81.5)	64 (60.4)	
**Perception on exposure risk toward HBV infection**			0.067
High risk	100 (76.9)	59 (59.0)	
Low risk	30 (23.1)	12 (40.0)	
**Perception on vaccine effectiveness**			0.508
Very effective	99 (76.2)	78 (78.8)	
Not very effective	31 (23.8)	24 (77.4)	
**Perception of vaccine safety**			0.351
Safe	114 (87.7)	64 (87.7)	
Not safe	16 (12.3)	7 (43.8)	
**Importance of vaccine to healthcare workers**			0.055
Yes	129 (99.2)	71 (55.0)	
No	1 (0.8)	0 (0.0)	

* Percentages represent the frequency of participants in each category out of the total participants. The percentages in the vaccination uptake represent the proportion of participants vaccinated within each category (row percentages).

### Uptake of hepatitis B vaccination among health laboratory practitioners

The overall uptake of the hepatitis B vaccine among study participants was 54.6% (71/130).

Although not statistically significant, we observed that female participants tended to have higher vaccine uptake, 63.6% (35/55), compared to males, 48.0% (36/75) (p = 0.077). Similarly, participants who perceived themselves as being at high risk of HBV exposure had a higher uptake rate of 59.0% (59/100) compared to those perceiving themselves at low risk (40.0%, 12/30) (p = 0.067). No significant difference was found in vaccine uptake based on participants’ age, years of work experience, perception of vaccine effectiveness, health facility, laboratory section, or perceptions of vaccine safety and importance among healthcare workers. “Table 1”

### Barriers to Hepatitis B vaccination and knowledge of recommended doses

Despite a high level of knowledge and positive perceptions, some participants remained unvaccinated or incompletely vaccinated. Among those who did not complete the vaccine series, the main reasons were missed appointments (48.4%, 15/31) and unavailability of the vaccine at their workplace (41.9%, 13/31). For those unvaccinated, the most common reason was a lack of opportunity to receive the vaccine, at 50% (14/28). Most participants were aware that the recommended hepatitis B vaccination schedule consists of three doses, as indicated by 94.1% (96/102) “Table 2”.

**Table 2:** Barriers to HBV vaccination and knowledge of recommended doses.

Variables	Frequency (*n*)	Percentage (%)
**Reasons for not completing full vaccination (*n* = 31)**		
Missed appointment	15	48.4
Not available in my working place	13	41.9
It is expensive can’t afford	3	9.7
**Reasons for not being vaccinated (*n* = 28)**		
Vaccine is not available in my working place	4	14.3
It is expensive	2	7.1
I’m concerned about side effects	8	28.6
I have not been offered the chance	14	50.0
**Number of recommended doses of Hepatitis B vaccine (*n* = 102)**		
Three doses	96	94.1
I don’t know	6	5.9

### Factors influencing hepatitis B vaccination uptake

In univariate analysis, none of the examined sociodemographic or occupational factors were statistically significantly associated with hepatitis B vaccination uptake. Females had a higher prevalence of vaccination compared to males (cPR = 1.421, 95% CI: 0.662-2.632, p = 0.427), although this association did not reach statistical significance.

Compared to participants aged 20–29 years, those aged 30–39 years (cPR = 1.116, 95% CI: 0.319–3.908, p = 0.864) and those aged ≥40 years (cPR = 2.129, 95% CI: 0.694–6.534, p = 0.187) showed no statistically significant differences in vaccination uptake.

Participants with more than two years of work experience had a higher prevalence of vaccination compared to those with less than two years (cPR = 1.767, 95% CI: 0.886–3.552, p = 0.106), but this association was not statistically significant. Similarly, high perceived risk of HBV exposure was not significantly associated with vaccination uptake (cPR = 1.475, 95% CI: 0.924–2.355, p = 0.143).

In multivariable analysis adjusting for age, none of the variables were independently associated with vaccination uptake. “Table 3”

**Table 3:** Univariate and multivariate analysis of factors influencing Hepatitis B vaccination uptake.

Variable	Category	Univariate analysis			Multivariate analysis		
		cPR	95% CI	p – value	aPR	95% CI	p – value
**Sex**	Male	Ref					
	Female	1.421	0.662-2.632	0.427	1.407	0.529-3.744	0.494
**Age group (years)**	20 – 29	Ref					
	30 – 39	1.116	0.319-3.908	0.864	1.082	0.307-3.815	0.903
	40+	2.129	0.694-6.534	0.187	1.891	0.595-6.007	0.280
**Work experience (years)**	Less than 2 years	Ref					
	More than 2 years	1.767	0.886-3.552	0.106	1.386	0.685-2.806	0.364
**Perception on exposure risk toward HBV infection**	Low risk	Ref					
	High risk	1.475	0.924-2.355	0.143	1.418	0.910-2.208	0.123

## Discussion

This study demonstrates that more than half of health laboratory practitioners (HLPs) in referral and tertiary hospitals in Dar es Salaam had completed the hepatitis B vaccination series. This uptake remains suboptimal given Tanzania’s high endemicity and the occupational risk inherent in laboratory practice. Although nearly all participants acknowledged the importance of vaccination, and the majority perceived the vaccine as safe and very effective, completion rates did not reflect this high awareness. Importantly, no sociodemographic, occupational, or perceptual factor independently predicted vaccination uptake in multivariable analysis. These findings suggest that structural determinants, rather than knowledge or individual characteristics, primarily explain incomplete vaccination in this high-risk professional group.

In this study, the full-dose hepatitis B vaccine uptake among laboratory practitioners was 54.6%, which is comparable to findings from Tanzania and across Africa. A 2015 survey at Muhimbili National Hospital in Dar es Salaam reported 56.2% uptake among staffs working in laboratory/theatre [16]. A Pan-African HCW survey conducted in 12 countries including Kenya, Uganda and Tanzania reported uptake of 49% among laboratory personnel [18]. These figures closely align with our estimate and suggest a persistent regional pattern of moderate uptake among laboratory cadres.

Globally, hepatitis B vaccination uptake among laboratory personnel varies widely across regions, with rates generally higher in middle- and high-income settings compared to low- and middle-income countries (LMICs). A study at a tertiary care center in India that disaggregated laboratory technicians from other HCW groups showed that 78.7% of laboratory technicians were vaccinated against hepatitis B, with 57.4% having completed the full three-dose schedule [19]. These figures are notably higher than the 39.5% three-dose completion observed among laboratory workers in Baghdad, Iraq, where 73.3% reported any vaccination but only 39.5% completed the full schedule [20].

Comparing these findings to our study, where 54.6% of laboratory practitioners in Dar es Salaam completed the full three-dose series, our coverage is substantially lower than estimates reported for Indian and some Middle Eastern laboratories (57.4–78.7%) but higher in completed series than the 39.5% found among Iraqi laboratory staff. This gradient likely reflects differences in institutional vaccination policies, occupational health infrastructure, and systematic follow-up of multi-dose schedules between settings [21].

Several contextual factors could explain these variations. In high-uptake settings such as India, mandatory or highly encouraged vaccination programs integrated into hospital occupational health systems likely promote better series completion. In contrast, in LMICs like Yemen and Iraq, where vaccination may be voluntary or dependent on individual initiative and sporadic vaccine supply, completion rates even among laboratory workers who are at high risk can remain incomplete. Moreover, the strong knowledge base among lab staff in Yemen and Iraq did not necessarily translate into completed protection, underscoring that knowledge alone does not ensure full vaccination uptake without consistent health system support and structured delivery of the three-dose regimen [21].

Overall, the global evidence indicates that laboratory staff vaccination uptake is heterogeneous, frequently above 60% in middle-income settings with stronger health systems but significantly lower where occupational vaccination is not mandated or systematically implemented. Our 54.6% completion rate places Dar es Salaam’s laboratory cohort in the middle of this global distribution, highlighting persistent opportunities to strengthen occupational health policy, consistent vaccine availability, and structured uptake monitoring to bridge the gap between awareness and complete immunization.

Despite the high level of knowledge regarding the recommended three-dose hepatitis B vaccination schedule (94.1%), a considerable proportion of laboratory practitioners in our study remained either unvaccinated or incompletely vaccinated, with the predominant barriers being missed appointments, lack of opportunity, and unavailability of the vaccine at the workplace. These findings strongly suggest that logistical and system-level constraints, rather than knowledge deficits, underlie incomplete uptake within this cadre. Similar patterns have been documented among laboratory staff in Yemen, which reported that although awareness of HBV risk and vaccine schedules was high among clinical laboratory workers, gaps in institutional vaccine provision and inconsistent occupational health implementation limited full uptake [21]. Likewise, in India, observed that incomplete vaccination among laboratory healthcare workers was frequently linked to missed doses and access-related issues rather than ignorance of recommended schedules [19]. Importantly, the prominence of “missed appointments” and “not being offered the chance” in our findings indicates missed opportunities within occupational health systems, particularly in environments where laboratory professionals face routine exposure to blood-borne pathogens. Taken together, these comparisons reinforce that, among laboratory professionals, knowledge of recommended dosing alone is insufficient to ensure completion of the vaccine series; structured workplace delivery systems, reminder mechanisms, and guaranteed vaccine availability appear to be more decisive in achieving full immunization coverage in this high-risk occupational group.

In this cohort of laboratory professionals, none of the examined sociodemographic or occupational factors including sex, age group, work experience, and perceived exposure risk were independently associated with hepatitis B vaccination uptake after multivariable adjustment, despite elevated crude prevalence ratios for females and more experienced staff. This pattern is consistent with cadre-specific evidence suggesting that, within laboratory settings, structural and institutional determinants may outweigh individual characteristics in shaping vaccination behavior. For example, a study among clinical laboratory staff in Yemen reported substantial HBV vaccination coverage but demonstrated that uptake was significantly associated with biosafety training and availability of written safety guidelines rather than basic demographics [21]. Similarly, among laboratory healthcare workers in India, observed marked differences in vaccination uptake across occupational roles within the laboratory workforce highlighting organizational and access-related disparities rather than age or sex as primary determinants [19]. Therefore, our findings reinforce the evidence that systemic enablers such as institutional vaccine provision, occupational health mandates, and biosafety infrastructure are more critical determinants of HBV vaccination uptake than individual sociodemographic in occupational groups with high baseline exposure.

This study provides valuable insight into hepatitis B vaccination uptake among laboratory practitioners in Dar es Salaam, contributing to the limited local data on occupational vaccine uptake. A key strength is the use of a structured data collection approach and verification of vaccination status, which enhances the accuracy of reported uptake. Additionally, including multiple hospitals improves the representativeness of the findings within the city’s healthcare setting. However, this study has several limitations. Although vaccination status was verified through documented records, the exclusion of participants without proof of vaccination may have introduced some degree of selection bias. In addition, the use of convenience consecutive sampling within hospitals, together with deviations from the intended probability proportional to size (PPS) allocation across sites, may have affected the representativeness of participants across facilities.

Furthermore, while the sample size met the minimum calculated requirement, it remains relatively modest, which may have limited the statistical power to detect some associations, as reflected by the wide confidence intervals observed. Consequently, the absence of statistically significant predictors should be interpreted with caution. These factors may also limit the generalizability of the findings beyond similar tertiary healthcare settings.

Finally, although selected knowledge and perception variables related to hepatitis B vaccination uptake were assessed, these were not explored in a comprehensive knowledge, attitude, and practice framework, which may limit a more in-depth understanding of behavioral determinants of vaccine uptake.

## Conclusion

Although more than half of the participants achieved full-dose coverage, nearly half were either partially vaccinated or unvaccinated, leaving a significant proportion at risk of HBV infection. No sociodemographic, occupational, or perceptual factors significantly predicted uptake; however, programmatic obstacles, such as missed appointments and inconsistent vaccine supply, remain substantial barriers to uptake. To improve protection of healthcare workers, hospitals should prioritize on-site vaccination services, ensure reliable vaccine availability, and adopt reminder systems or mandatory pre-employment vaccination policies. These measures would close existing gaps and strengthen progress toward the elimination of HBV targets.

## Supporting information

S1 Data Data set underlying the findings of this study. All relevant data underlying the findings of this study are provided within the manuscript and its supporting information files.
(SAV)

S1 File STROBE checklist. (DOCX)
