# Supplementary material for: Uptake and determinants of Hepatitis B vaccination among health laboratory practitioners in tertiary hospitals in Dar es Salaam, Tanzania: A cross-sectional study
Source: PLOS Glob Public Health. 2026 Apr 27;6(4):e0005662. doi: 10.1371/journal.pgph.0005662 (PMC13119911; doi:10.1371/journal.pgph.0005662)
Supplement: S1 File — (DOCX) [file pgph.0005662.s002.docx]

# STROBE Checklist for Cross-sectional Studies

Manuscript title: **Uptake and Determinants of Hepatitis B Vaccination among Health Laboratory Practitioners in Tertiary Hospitals in Dar es Salaam, Tanzania: A Cross-Sectional Study.**

| Item No. | Recommendation | Reported on Page/Section |
| --- | --- | --- |
| 1a | Indicate the study’s design with a commonly used term in the title or the abstract | Title, Abstract |
| 2 | Explain the scientific background and rationale for the investigation being reported | Introduction, p.4-5 |
| 3 | State specific objectives, including any prespecified hypotheses | Introduction, p.4-5 |
| 4 | Present key elements of study design early in the paper | Methods, Study design, p.6 |
| 5 | Describe the setting, locations, and relevant dates, including periods of recruitment | Methods, Setting, p.6 |
| 6a | Give the eligibility criteria, and the sources and methods of participant selection | Methods, Participants, p.7-6 |
| 7 | Clearly define all outcomes, exposures, predictors, potential confounders | Methods, Variables, p.8-9 |
| 8 | For each variable of interest, give sources of data and details of methods of assessment | Methods, Data collection, p.8 |
| 9 | Describe any efforts to address potential sources of bias | Methods, Bias, p.9 |
| 10 | Explain how the study size was arrived at | Methods, Sample size, p.7 |
| 11 | Explain how quantitative variables were handled in the analyses | Methods, Statistical analysis, p.8 |
| 12 | Describe all statistical methods, including those used to control for confounding | Methods, Statistical analysis, p.8-9 |
| 13a | Report numbers of individuals at each stage of study (e.g., numbers eligible, included) | Methods, Participant flow, p.7 |
| 14 | Give characteristics of study participants (e.g., demographic, clinical, social) | Results, p.9 |
| 15 | Report numbers of outcome events or summary measures | Results, Table 1-2, p.10-13, |
| 16a | Give unadjusted estimates and, if applicable, confounder-adjusted estimates and precision | Results, Table 3, p.14-15 |
| 17 | Report other analyses done (e.g., subgroup analyses, sensitivity analyses) | Results, Additional analysis, p.10 |
| 18 | Summarise key results with reference to study objectives | Discussion, p.15 |
| 19 | Discuss limitations of the study | Discussion, p.18 |
| 20 | Give a cautious overall interpretation of results considering objectives and limitations | Discussion, p.18 |
| 21 | Discuss the generalizability (external validity) of the study results | Discussion, p.17-18 |
| 22 | Give the source of funding and the role of the funders | Funding statement, p.19 |
